# Increased IL-12p70 Levels in Intraoperative Pericardial Fluid Are Predictive of Postoperative Atrial Fibrillation Onset after Coronary Artery Bypass Surgery

**DOI:** 10.31083/j.rcm2505166

**Published:** 2024-05-13

**Authors:** Yuhua Liu, Enzehua Xie, Yunxiao Yang, Zhongyi Han, Cuntao Yu, Kun Hua, Xiubin Yang

**Affiliations:** ^1^Department of Cardiovascular Surgery, Beijing Anzhen Hospital, Capital Medical University, 100029 Beijing, China; ^2^Department of Cardiovascular Surgery, Fuwai Hospital, State Key Laboratory of Cardiovascular Disease, Chinese Academy of Medical Sciences & Peking Union Medical College/National Center for Cardiovascular Diseases, 100037 Beijing, China

**Keywords:** postoperative atrial fibrillation, IL12p70, intraoperative pericardial fluid, magnetic luminex assay

## Abstract

**Background:**

Postoperative atrial fibrillation (POAF) is a frequent complication of heart surgery, prolonging hospital stays, as well as increasing morbidity and mortality rates. While previous studies have investigated the determinants influencing atrial fibrillation (AF) following heart surgery, the specific risk factors contributing to POAF occurrence after coronary artery bypass graft surgery (CABG) are not well understood. Here we used the human magnetic Luminex assay to assess whether biomarkers, particularly cytokines, within intraoperative pericardial fluid could serve as predictive markers for POAF onset among CABG individuals.

**Methods:**

In this study we identified 180 patients who underwent CABG with no atrial arrhythmia history. The human magnetic Luminex assay was used to quantify the levels of 36 cytokines in pericardial fluid samples collected during the surgery. The occurrence of POAF was continuously monitored, using both postoperative electrocardiograms and telemetry strips, until the time of discharge.

**Results:**

In our cohort of 124 patients, POAF was observed in 30 patients, accounting for 24.19% of the study population. These patients exhibited significantly higher levels of interleukin (IL)-12p70 in their intraoperative pericardial fluids compared to those with normal sinus rhythms (SR, *p* < 0.001). Subsequently, IL-12p70 was found to be an independent risk factor for POAF, and receiver operating characteristic (ROC) analysis established a cut-off threshold for predicting POAF onset of 116.435 pg/mL, based on the maximum Youden index (area under the curve: 0.816).

**Conclusions:**

this study establishes a significant association between elevated IL-12p70 levels in intraoperative pericardial fluid and the risk of POAF, particularly when IL-12p70 concentrations exceed the identified cut-off value of 116.435 pg/mL. These findings suggest that IL-12p70 levels could potentially be utilized as a predictive biomarker for the onset of POAF in patients undergoing CABG. This marker may aid in the early identification and management of patients at heightened risk for this complication.

## 1. Introduction

Post-operative atrial fibrillation (POAF) is a common complication following various surgical procedures, with its incidence varying depending on the type of procedure. It typically manifests within the first two to four days following surgery [1, 2]. In vascular or major colorectal surgeries, POAF occurs in 5–20% of cases, whereas its incidence rises to 20–30% after non-cardiac thoracic surgeries, and 20–50% following cardiac procedures [3, 4, 5, 6]. Notably, in coronary artery bypass graft surgery (CABG), POAF’s incidence ranges from 25–40% [7], and its occurrence is independent of whether the surgery involves cardiopulmonary bypass (CPB) [8].

Furthermore, POAF significantly impacts patient outcomes and healthcare costs. It not only increases the risk of stroke, morbidity, and mortality, but also prolongs hospital stays and overall costs [9]. Patients with POAF face four- to fivefold higher risk for recurrent atrial fibrillation (AF) in the following five years [10, 11]. Additionally, POAF is associated with increased short- and long-term morbidities and mortalities in other diseases, such as congestive heart failure, renal insufficiency, and serious infections. These comorbidities often result from extended hospitalizations or myocardial infarction [12, 13].

Various factors have been identified as increasing the risk for POAF, including age, AF history, sex, previous valvular cardiac surgery, hypertension, diabetes, decreased left ventricular ejection fraction, enlarged left atrium, and being overweight [13, 14, 15]. Despite recognizing these risk factors, predicting POAF in specific individuals remains challenging, as does fully understanding the underlying mechanisms of its occurrence. One possible etiology is presence of pre-operative structural substrates that enhance the risk of atrial electrical re-entry, leading to higher incidence for post-operative cardiac physiological perturbations [2]. This hypothesis is supported by studies indicating a shared pathogenesis pathway between pre-existing atrial fibrillation (pre-AF) and POAF [3]. However, this pathway is complicated by predisposing factors such as inflammation, sympathetic stimulation, and cardiac ischemia, all of which are present among CABG individuals with POAF, and increase their vulnerability for AF induction and maintenance [2]. Given these complexities, further research is crucial to elucidate the pathophysiology of POAF, especially in relation to pre-AF conditions. Such understanding is vital for developing targeted preventive and therapeutic strategies.

Research has shown that individuals susceptible to POAF individuals have been found to possess exhibit distinct plasma protein and metabolite levels compared to those who do not develop POAF. pecifically, POAF onset has been associated with elevated glutathione peroxidase 3 (GPX3), B-type natriuretic peptide (BNP) and cholesteryl ester transfer protein (CETP), as well as decreased phospholipid transfer protein (PLTP) and apolipoprotein-C3 (APOC3) [16, 17]. However, these studies only examined pre-operative plasma, which serves as a reflection of the overall body state, rather than the heart specifically. Therefore, determining metabolite level differences between POAF and non-POAF patients, specifically related to alterations in cardiac functioning, is of great importance. As the heart and surrounding tissues are noted to produce various physiologically active substances, identifying and measuring the levels of pericardial factors associated with increased POAF risk, prior to CABG surgery could be pivotal. Such an approach not only holds potential for developing diagnostic and treatment strategies but also contributes to a deeper understanding of POAF pathogenesis.

Recent clinical studies have bolstered the approach of focusing on pericardial fluid for POAF biomarkers. These studies have identified specific biological indicators in pericardial fluid that are elevated in comparison to plasma levels, providing insights into POAF pathogenesis. For instance, Manghelli *et al*. [18] discovered a significant association between mitochondrial DNA in pericardial fluid and POAF progression. Similarly, Liu *et al*. [19] established a causal relationship between pericardial interleukin-6 (IL-6) levels and POAF in mice, noting that IL-6 stimulates profibrotic pathways in cardiac myocytes via the phosphorylated-signal transducers and activators of transcription 3 (p-STAT3) mechanism, particularly in the early post-surgery phase. All of these findings, therefore, indicate that inflammatory mechanisms and mediators present in pericardial fluid may play significant roles in POAF onset [20], suggesting that pericardial fluid biomarker analysis could serve as a viable approach for predicting cardiac disease occurrence, such as POAF.

In this study, we aimed to investigate whether mediators in pericardial fluids could serve as biomarkers for increased POAF susceptibility in CABG patients. We also aimed to identify early and precise diagnosis markers for POAF and provide novel treatment avenues for POAF prevention.

## 2. Materials and Methods

### 2.1 Participants and Study Design

This study consisted of a retrospective analysis of prospectively-collected pericardial fluid from patients with no history of atrial arrythmia, undergoing elective CABG, was conducted at Beijing An-Zhen Hospital. Pericardial fluid was collected intraoperatively from February 2022- October 2022, with samples subsequently being frozen for analysis. Patient exclusion criteria were as follows: CABG combined with other surgical procedures, such as valve replacement/repair, preoperative arrhythmia/pre-AF, lack of informed written consent, and post-surgery fatalities.

After applying exclusion criteria, fluid was collected from 124 patients, of which after CABG, 30 (24.19%) developed POAF, and 94 (75.81%) retained proper sinus rhythm (SR). Out of those 124 patients, in accordance with recommendations from Lonjon *et al*. [21], 56 were chosen for the study by matching the most pertinent clinical parameters, including age, sex, and potential clinical features, as well as routine biochemical parameters. To ensure balanced cohorts for POAF and SR patients pre-operation, propensity score matching was conducted, matching patients in a 1:1 ratio using a standard caliper width of 0.2 on the propensity score. This process resulted in 20 participants being assigned to each cohort (Table 1).

**Table 1.** **Preoperative characteristics of patients with POAF and SR**.

Variable	Unmatched			1:1 PSM		
	POAF	SR	*p*-value	POAF	SR	*p*-value
	(N = 30)	(N = 94)		(N = 20)	(N = 20)	
Age (years)	64.06 ± 8.40	63.45 ± 4.68	0.616	63.84 ± 7.84	63.50 ± 7.16	0.887
Male sex	24 (80.00)	75 (79.70)	0.360	15 (75.00)	15 (75.00)	1.000
BMI (kg/$m^{2}$)	26.25 ± 3.01	25.62 ± 2.71	0.283	25.66 ± 3.34	25.63 ± 2.59	0.975
Hypertension	24 (80.00)	58 (61.70)	0.020	14 (70.00)	16 (80.00)	0.265
Diabetes mellitus	16 (53.33)	41 (43.62)	0.239	13 (65.00)	15 (75.00)	0.593
TG (mmol/L)	1.64 (1.09, 1.81)	1.60 (1.18, 1.83)	0.826	1.58 (1.04, 1.92)	1.60 (1.09, 1.94)	0.866
TC (mmol/L)	3.99 ± 0.87	4.10 ± 0.93	0.568	4.00 ± 0.98	4.19 ± 0.88	0.523
HDL-C (mmol/L)	0.97 ± 0.19	1.05 ± 0.25	0.110	1.00 ± 0.22	1.00 ± 0.28	1.000
LDL-C (mmol/L)	2.13 ± 0.70	2.32 ± 0.71	0.203	2.17 ± 0.78	2.27 ± 0.73	0.678
Lp(a) (nmol/L)	33.10 (6.33, 100.10)	29.60 (4.33, 102.20)	0.949	37.80 (10.65, 98.03)	44.40 (16.60, 117.50)	0.332
LVEF (%)	59.43 ± 7.17	60.60 ± 4.65	0.300	60.98 ± 6.74	62.20 ± 6.07	0.551
LAD (mm)	37.35 ± 3.12	35.23 ± 4.03	0.009	36.66 ± 3.89	35.79 ± 3.43	0.458
Logistic EuroSCORE II	5.87 ± 2.09	5.26 ± 1.38	0.068	5.81 ± 2.08	5.52 ± 1.65	0.528
Duration of surgery (hours)	4.09 ± 0.90	4.25 ± 0.85	0.378	4.18 ± 0.72	4.20 ± 0.71	0.930
Number of coronary grafts	3.57 ± 0.77	3.30 ± 0.70	0.075	3.47 ± 0.63	3.42 ± 0.80	0.817

POAF, postoperative atrial fibrillation; SR, sinus rhythms; LVEF, left ventricular ejection fraction; TG, triglyceride; TC, total cholesterol; LDL-C, low-density lipoprotein cholesterol; Lp(a), Lipoprotein-a; HDL-C, high-density lipoprotein cholesterol; BMI, body mass index; LAD, left atrial diameter; PSM, propensity score matching.

Routine biochemical parameters were then assessed using the Hitachi-7600 (Tokyo, Japan) chemical analyzer in the Biochemical Laboratory department, with quality control being conducted using blinded quality control samples. Beijing An-Zhen Hospital’s Medical Ethics Committee approved the research protocol, which adhered to the Declaration of Helsinki. All patients provided written informed consent.

### 2.2 Collecting and Storing Pericardial Fluid

Anesthesia was administered for CABG surgery, followed by endotracheal intubation with a single lumen and median sternotomy. The procedure was conducted on beating hearts, without CPB, and it, along with perioperative care, was identical for each patient. To maximize surgical timeframes and eliminate confounding variables, all pericardial fluid specimens were collected via suction through a sterilized, disposable syringe immediately during intraoperative opening of the pericardium, but prior to receiving heparin injections. A minimum of 1 mL pericardial fluid was collected from each patient, and care was taken to ensure that no blood was mixed in during the collection process. After collecting the fluid, samples were placed in sterile containers, and immediately stored at –80 °C until testing.

### 2.3 Luminex Assays

Magnetic Luminex® Assays are an antibody microarray based on magnetic beads that allows simultaneous quantification of antibody levels in a sample [22]. The presence of POAF-associated biomarkers within pericardial fluid was detected using the Luminex (R&D Systems, Inc., Minneapolis, MN, USA) panel. Prior to conducting these assays, we ensured that all pericardial fluid samples were diluted to the appropriate concentrations, so that the cytokine concentration questions would be within the dynamic detectability range. Furthermore, all standards and samples were performed in duplicate, according to the manufacturer’s guidelines. A total of 36 cytokines involved in inflammation, fibrosis, and atrial remodeling were thus analyzed: tumor necrosis factor (TNF)-α, glycoprotein (gp) 130, platelet-derived growth factor (PDGF)-BB, chemokine (C-C motif) ligand 1 (CCL1), IL-8, fibroblast activation protein (FAP)-α, IL-10, fatty acid binding protein 4 (FABP4), angiopoietin-2, chemokine (C-X-C motif) ligand 13 (CXCL13), interferon (IFN)-γ, IL-1ra, CCL18, IL-12p70, CCL3, CCL4, insulin-like growth factor binding protein 1 (IGFBP-1), CXCL16, IL-17, adiponectin, CCL26, granulocyte-macrophage colony-stimulating factor (GM-CSF), fibroblast growth factor 2 (FGF2), cardiac troponin, leptin, CXCL5, osteopontin, thrombopoietin, fibronectin, IL-1β, oncostatin M, CXCL11, angiopoietin-1, IL-6, cluster of differentiation 40 (CD40) ligand and CCL17.

### 2.4 Evaluation and Treatment of POAF after CABG

AF has been observed to be able to occur during (perioperative) or after (POAF) heart surgery procedures. POAF was defined as newly-onset occurrences of AF during the time period immediately post-operation and has been considered a medically significant issue. However, POAF could vary from asymptomatic and self-terminating bouts to AF lasting for at least 30 seconds [23], which was the main outcome observed among our patients.

Heart rhythms were constantly observed for all hospitalized patients in this study using either bedside arrhythmia monitors or telemetry. Suspected AF were verified using an extra 12-lead electrocardiogram, and individuals diagnosed with POAF were provided with 2 management options: rhythm or rate control. Rhythm control was recommended for patients who were hemodynamically unsteady, highly symptomatic, possessing anticoagulant contraindication, or were already subject to electrical cardioversion, amiodarone, or both, while rate control was preferred for all other patients, as those would spontaneously return to normal SR within 6 weeks post-discharge. Additionally, all patients with AF >1–2 days were prescribed anticoagulants unless contraindicated.

### 2.5 Determining the Predictive Variables for Increased POAF Risk and Statistical Analyses

All statistical analyses were conducted using SPSS (25.0.0.0, IBM Corp., Armonk, NY, USA) and R (4.1.3, R Foundation for Statistical Computing, Vienna, Austria) software. To identify the variables most strongly associated with increased POAF risk, both uni- and multivariate logistic regression analyses were conducted. Receiver operating characteristic (ROC) curve analysis was then carried out to determine the most optimal cut-off point for those variables. Net clinical benefits for the identified variables was determined using decision curve analysis (DCA).

In order to account for any confounding variations for variables between POAF and SR cohorts, a 1:1 optimum matching technique was employed. In the case of normal distribution variables, the mean and standard deviation (SD) are presented; otherwise, the median and interquartile range are presented. Continuous variables with normal distribution were analyzed using the Students’ *t* test, while those with non-normal distribution were analyzed using the Wilcoxon rank-sum test. Yates adjustment was used to compare categorical variables.

## 3. Results

### 3.1 Baseline Clinical Features of the Study Population

**Fig. 1.**
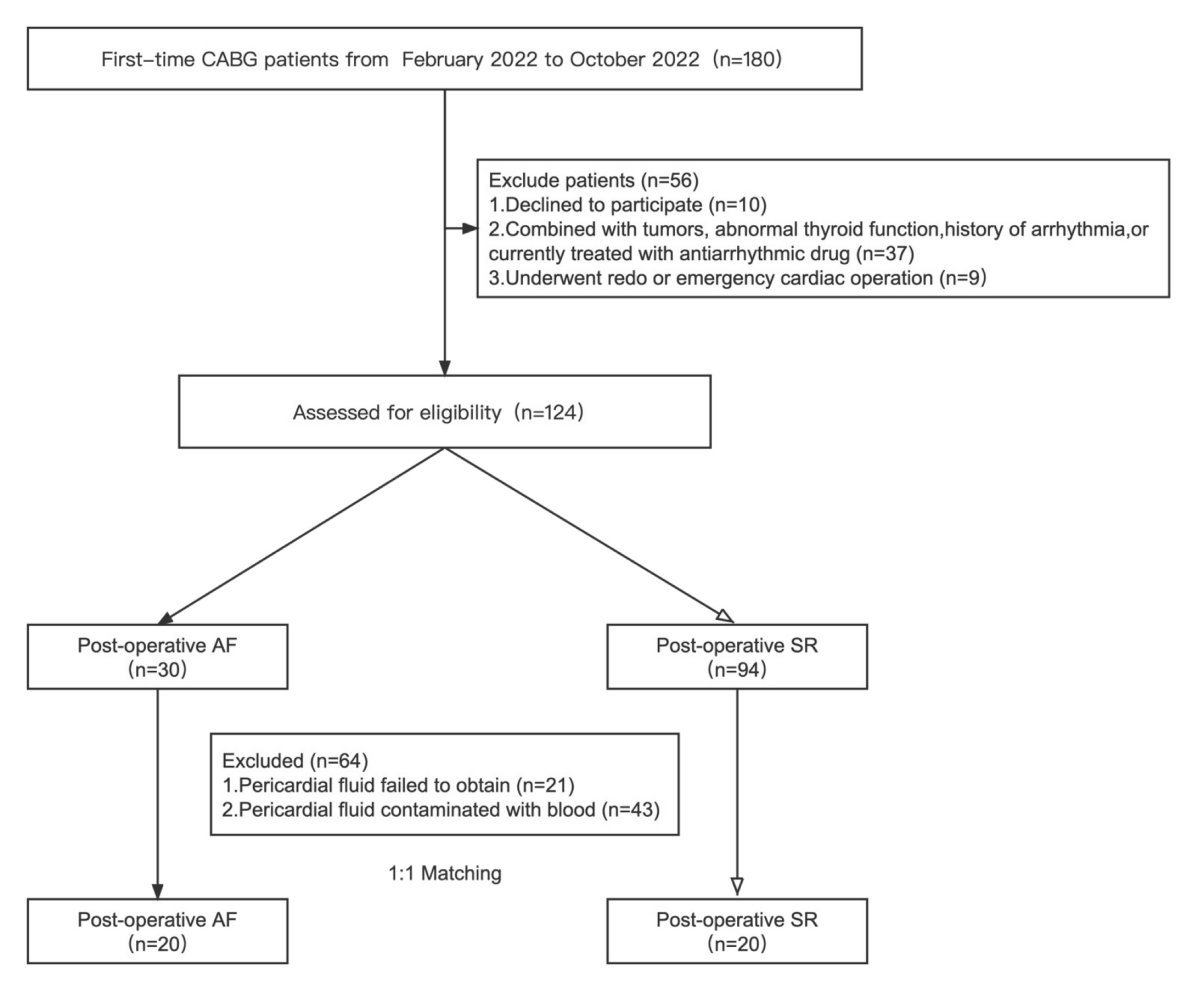
**Flow chart of the study**. CABG, coronary artery bypass grafting; SR, sinus rhythm; AF, atrial fibrillation.

This study involved 180 patients undergoing their first CABG surgery. After applying exclusion criteria, 124 patients were included for monitoring postoperative heart rhythms. Of these, 94 (75.81%) maintained SR, and 30 (24.19%) developed POAF (Fig. 1), which occurred between days 1–5 post-surgery (average 2.25 days). All 30 POAF patients were able to regain normal SR after being administered intravenous antiarrhythmic agents. From patients with SR or POAF, 2 cohorts were established. We excluded 64 patients due to challenges in obtaining pericardial fluid (n = 21) or pericardial fluid contamination with blood (n = 43). Of the 64 patients, this procedure resulted in 8 POAF patients and 56 SR patients. After 1:1 propensity score matching (PSM), we established two equally sized cohorts of 20 patients each, designated as the SR and POAF cohorts. Before PSM, the POAF cohort displayed a higher prevalence of hypertension and larger left atrial diameters compared to the SR cohort (Table 1).

### 3.2 Pericardial Fluid IL-12p70 was an Independent Risk Factor for POAF

**Fig. 2.**
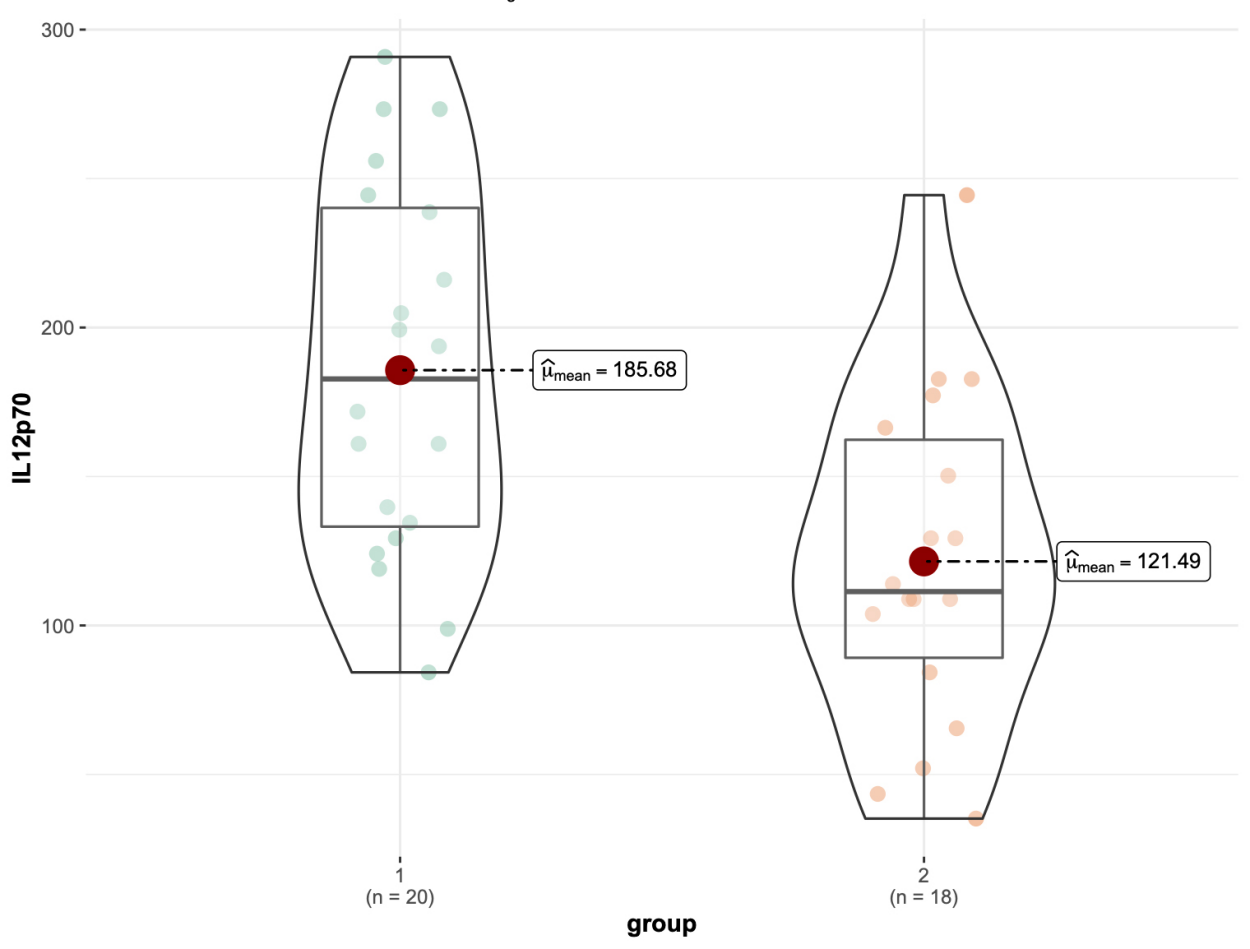
**Comparison of Intraoperative pericardial fluid levels**. µmean, mean; IL-12p70, interleukin-12p70.

In this study, the Magnetic Luminex test was used to assess the relationship between 36 cytokines in pericardial fluid and the incidence of POAF. Notably, IL-12p70 levels were found to be significantly higher in the POAF patient cohort (n = 20) compared to the SR (n = 18, 2 outliers were removed) cohort (*p* < 0.001; Fig. 2). Uni- and multivariate logistic regression analyses were then conducted on those 36 pericardial fluid cytokines, to determine their association with POAF onset risk. Statistically significant (*p* < 0.05) factors identified from univariate logistic regression analysis were then incorporated into the multivariable logistic regression model, which corrected for factors such as age, sex, body mass index (BMI), hypertension, presence of diabetes, left atrial diameter, left ventricular ejection fraction, EuroSCORE II, and number of grafts. After adjusting for these factors, IL-12p70 emerged as the sole cytokine independently associated with an increased risk of POAF. Specifically, a rise in pericardial fluid IL-12p70 levels was linked to a heightened POAF risk (odds ratio [OR] = 1.201; 95% confidence interval [CI], 1.001–1.510; *p* = 0.014; Table 2).

**Table 2.** **POAF univariate and multiple logistic regression models**.

	Univariate model			Multiple model		
	OR	95% CI	*p*-value	OR	95% CI	*p*-value
IL12p70	1.019	1.001–1.330	0.025	1.201	1.001–1.510	0.014
Age (years)	0.940	0.883–1.052	0.450	1.105	0.243–1.334	0.879
Sex	1.000	0.255–3.926	1.000	1.015	0.255–1.430	0.725
BMI (kg/$m^{2}$)	1.049	0.873–1.269	0.538	2.826	1.129–8.320	0.081
Hypertension	1.575	0.618–5.690	0.267	1.412	0.126–3.345	0.507
Diabetes mellitus	0.741	0.263–2.147	0.593	1.192	0.878–1.631	0.272
LVEF (%)	1.102	0.934–1.275	0.957	0.996	0.982–1.010	0.564
LAD	1.275	1.130–5.251	0.525	0.970	0.920–1.016	0.602
EuroSCORE II	0.880	0.830–0.952	0.035	1.011	0.951–1.047	0.898
Number of grafts	3.124	1.002–4.783	0.323	2.808	1.010–4.430	0.059

POAF, postoperative atrial fibrillation; OR, odds ratio; BMI, body mass index; LVEF, left ventricular ejection fraction; LAD, left atrial diameter; IL, interleukin.

### 3.3 The Determination of Optimal IL-12p70 Cut-Off Levels via ROC Analysis

**Fig. 3.**
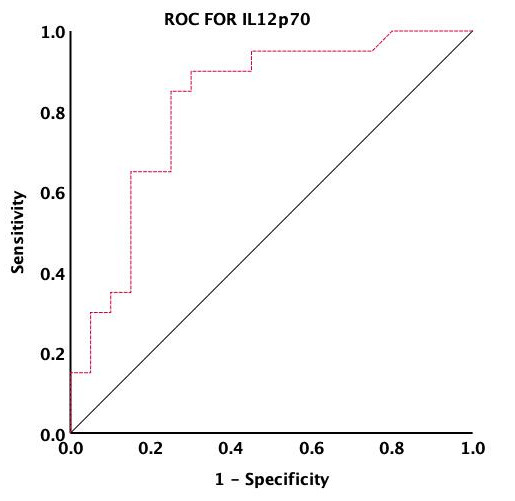
**Identification of the IL-12p70 cutoff point through ROC curve analysis**. The calculated cutoff point used the maximum Youden index (Youden index = sensitivity + specificity – 1). ROC, receiver operating characteristic; IL-12p70, interleukin-12p70.

To determine the cut-off point for IL-12p70 levels being indicative of increased POAF risk, we employed ROC curve analysis using the “pROC” package in R software. The optimal threshold value was determined based on the maximal Youden index (Youden index = sensitivity + specificity – 1), pinpointing 116.435 pg/mL (specificity: 57.0 %, sensitivity: 95.0%) as the maximal ROC-optimized cutoff value, with an area under the curve (AUC) of 0.816 (Fig. 3). This value was validated using both uni- and multivariate logistic regression analyses. The variable new_IL-12p70, representing IL-12p70 levels above 116.435 pg/mL, was found to be statistically significant in univariate (*p* = 0.00336), and multivariate (*p* = 0.00975) models. These findings underscore that patients with IL-12p70 levels above 116.435 pg/mL were more likely to develop POAF. Therefore, prioritizing these individuals in developing prevention and treatment strategies for POAF is crucial.

**Fig. 4.**
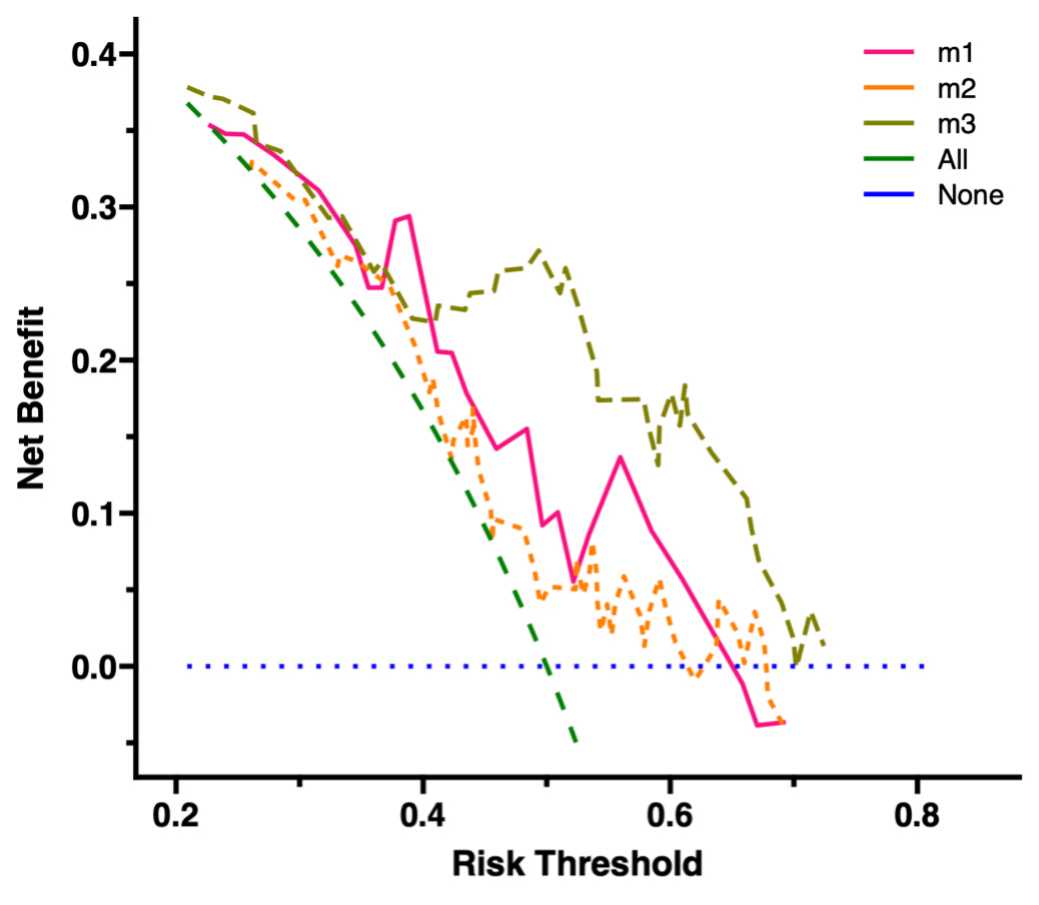
**Evaluation of clinical net benefit using DCA**. Models: m1: IL-12p70; m2: Other Clinical Characteristics; m3: Combined IL-12p70 and Clinical Characteristics. All, reference line 1. None, reference line 2. DCA, decision curve analysis; IL-12p70, interleukin-12p70.

The clinical utility of IL-12p70 levels above 116.435 pg/mL was then investigated with DCA, using the three models: (1) IL-12p70, (2) Other clinical characteristics, and (3) IL-12p70 + clinical characteristics. Results showed that model 3 offered the highest clinical utility, surpassing both models 1 and 2. This suggests that the threshold of IL-12p70 >116.435 pg/mL yielded greater benefits for predicting POAF, compared to other clinical characteristics alone. However, the combination of IL-12p70 and clinical characteristics was even more beneficial than for IL-12p70 alone (Fig. 4).

## 4. Discussion

In this study, we established a correlation between the presence of IL-12p70 cytokines and the onset of POAF following CABG. More specifically, POAF patients had significantly higher IL-12p70 levels, compared to those with normal SR. Moreover, we identified a critical threshold for IL-12p70, set at >116.435 pg/mL, using ROC curve analysis. This threshold was also confirmed to have clinical relevance through DCA. These findings suggest the potential of using IL-12p70 levels as a diagnostic marker for identifying CABG patients at heightened risk of developing POAF. By monitoring and potentially treating those with pericardial fluid IL-12p70 levels exceeding 116.435 pg/mL, this approach could significantly enhance post-surgery POAF management.

To the best of our knowledge, this study was the first to highlight the connection between IL-12p70 levels and POAF, though multiple previous studies have illustrated the presence of correlations between various inflammatory factors and POAF risk [24]. These studies, though, primarily focused on patient plasma, in which preoperative plasma samples were examined to measure the levels of various metabolites, and their association with POAF. However, the utility of these studies in pinpointing POAF risk remains constrained by inconsistent outcomes; findings linking specific metabolites to POAF risk in one study often do not translate to others [24]. This inconsistency between studies regarding plasma biomarkers may be due to the presence of local inflammation affecting the atria, thus diminishing their effectiveness in accurately predicting POAF risk.

Owing to these limitations of plasma proteins and metabolites as biomarkers for POAF risk, alternative sources, such as pericardial fluid proteins and metabolites, have become the subject of great interest. In a notable prospective study by Nakamura *et al*. [25], pericardial fluid was collected from 42 consecutive individuals receiving CABG to investigate the relationship between atrial and natriuretic peptide concentrations and POAF onset. They found that AF occurred in 9/42 (21%) patients receiving CABG, and that pericardial fluid BNP levels were independently associated with POAF occurrence [25]. These results were in line with the findings from our study, in which pericardial fluid BNP was higher in the POAF cohort versus that of SR. However, the increase in our study was not statistically significant, possibly owing to the smaller cohort sample size. It should be noted, though, that this lack of statistical significance is also supported by Manghelli *et al*. [18], who analyzed 36 pericardial fluid intraoperative cytokines and BNP, and found no significant relationship between their levels and POAF occurrence. This observation was also supported by our findings, in which intraoperative pericardial fluid BNP levels were not related to POAF onset.

The pathophysiology of AF is multifaceted, encompassing a range of factors, including inflammation, atrial remodeling, myocardial ischemia, and activation of the autonomic nervous system [2, 26]. These elements, along with specific atrial substrates, are believed to contribute to the development of POAF, increasing the susceptibility of patients to both the induction and maintenance of the condition. Accumulating evidence underscores the significance of inflammation in the etiology of POAF, with earlier studies indicating that inflammation may alter atrial conduction, potentially triggering POAF pathogenesis [27]. In a particular prospective study, elevated levels of IL-6 were detected in the pericardial drainage of patients with POAF, hinting at a surgery-induced intracardiac inflammatory microenvironment that could lead to transient POAF [19].

Contrasting these findings, our study, which analyzed intraoperative pericardial fluid for various inflammatory factors, revealed that only IL-12p70 levels significantly increased in POAF compared to normal SR conditions. The immunopeptide IL-12p70, is a heterodimeric cytokine composed of p40 and p35 subunits, is secreted by macrophages and dendritic cells [28]. It plays an essential role in stimulating natural killer (NK) cells, promoting their differentiation and growth, and is crucial for interferon-gamma (IFN-γ) production by NK cells and Th1 lymphocytes [29]. Furthermore, IL-12p70 enhances the expression of perforin, granzymes, and adhesion molecule expression, thereby augmenting the cytotoxicity of ${\mathrm{CD8}}^{+}$ T and NK cells [30]. Additionally, the antigen presentation capabilities of macrophages and dendritic cells are enhanced by IL-12p70 [31]. Collectively, these findings suggested that inflammation modulated by IL-12p70 is linked to the development of coronary artery disease [32, 33]. However, the specific details regarding the role of IL-12p70 in the inflammatory process are still largely undefined, though our findings suggest that inflammatory cytokines were already present within the pericardial fluid of coronary heart disease patients prior to CABG, and this pre-existing inflammation may contribute to POAF onset. Nevertheless, further studies are needed to fully elucidate the exact role of IL-12p70 in POAF.

There are some limitations in our study, one of which was the difficulty of collecting pericardial fluid, resulting in small sample sizes. This limitation might affect the robustness of our conclusions. Despite the small sample size, it was sufficient to detect significant differences in pericardial fluid cytokine levels between POAF and normal SR individuals. Furthermore, preoperative data of patients were also matched 1:1 between POAF and normal SR cohorts to adjust for confounding factors. Nevertheless, future studies with larger sample sizes are required to validate our findings and address the underlying basis behind the IL-12p70 increase in POAF.

## 5. Conclusions

In conclusion, POAF patients, compared to those with normal SR, had increased intraoperative pericardial fluid IL-12p70 levels after CABG. This association between increased IL-12p70 and POAF suggests a heightened risk of developing the disease, possibly due to increases in inflammation, fibrosis, and atrial remodeling-related cytokines, along with the presence of a pre-existing susceptible atrial substrate. Notably, the relationship between higher IL-12p70 and POAF was particularly evident at IL-12p70 levels exceeding 116.435, establishing this threshold as a potential predictive marker for POAF onset in future CABG patients. Consequently, IL-12p70 could serve as a valuable biomarker for assessing increased POAF susceptibility, particularly as pericardial fluid could be collected during open-heart surgery for CABG.

## Data Availability

All data points generated or analyzed during this study are included in this article and there are no further underlying data necessary to reproduce the results.

## Supplementary Material

Supplementary material associated with this article can be found, in the online version, at https://doi.org/10.31083/j.rcm2505166.
